# Phenotypic Diversity of *Cryptococcus neoformans* var. *neoformans* Clinical Isolates from Localized and Disseminated Infections

**DOI:** 10.3390/microorganisms10020321

**Published:** 2022-01-29

**Authors:** Zena M. Birkenfeld, Nikita Dittel, Thomas Harrer, Christoph Stephan, Albrecht F. Kiderlen, Volker Rickerts

**Affiliations:** 1 Department of Mycotic and Parasitic Agents and Mycobacteria, Robert Koch Institute, 13353 Berlin, Germany; zenabirkenfeld@dotlab.de (Z.M.B.); DittelN@rki.de (N.D.); KiderlenA@rki.de (A.F.K.); 2 Medizinische Klinik 3, Sektion Klinische Infektiologie und Immundefizienz, Universitätsklinikum Erlangen, Friedrich-Alexander-University Erlangen-Nürnberg, 91054 Erlangen, Germany; Thomas.Harrer@uk-erlangen.de; 3 Medizinische Klinik 2, Infektiologie und Therapie der HIV Infektion, Universitätsklinikum Frankfurt, 60596 Frankfurt am Main, Germany; C.Stephan@em.uni-frankfurt.de

**Keywords:** cryptococcosis, *Cryptococcus neoformans*, *Galleria mellonella*, *Acanthamoeba castellanii*, virulence factors

## Abstract

*Cryptococcus neoformans* var. *neoformans* is the second most prevalent agent of cryptococcosis in central Europe. Infections mostly present with localized skin and disseminated infections. Previous studies did not find these presentations to be determined by the fungal genotype as detected by multilocus sequence typing (MLST). However, phenotypic fungal traits may impact clinical presentation. Here, we studied the growth and virulence factors of *C. neoformans* var. *neoformans* isolates from disseminated and localized infections and an environmental isolate. We used coincubation with *Acanthamoeba castellanii* and the *Galleria mellonella* infection model to identify phenotypic characteristics potentially associated with clinical presentation. Clinical isolates of *C. neoformans* var. *neoformans* present a substantial phenotypic variability. Median survival of *G. mellonella* varied between 6 and 14 days. *C. neoformans* var. *neoformans* isolates from disseminated infections showed stronger melanization and larger capsules. They demonstrated superior uptake into an amoeba and increased cytotoxicity for the amoeba. Differences of strains from localized and disseminated infections in coincubation with amoeba are in line with the importance of phagocytes in the pathogenesis of disseminated cryptococcosis. Phenotypic traits and non-vertebrate infection models may help understand the virulence potential of *C. neoformans* var. *neoformans* isolates.

## 1. Introduction

Cryptococcosis is the most prevalent fungal infection of the central nervous system. Currently, the nomenclature of the agents of cryptococcosis is being reevaluated [1,2]. Here, we adhere to the nomenclature using *Cryptococcus neoformans* species complex. *C. neoformans* var. *grubii* is the most prevalent agent isolated from AIDS patients with meningoencephalitis worldwide [3]. Several virulence factors including the polysaccharide capsule and production of melanin were characterized. Although clinical isolates differ in these phenotypes, their contribution to clinical illness is poorly understood [4].

In Europe, up to 20% of human cryptococcosis cases are caused by *C. neoformans* var. *neoformans* [5,6]. Infection models generally suggest a lower virulence of *C. neoformans* var. *neoformans* strains as compared to *C. neoformans* var. *grubii* [7]. These infections may present with diverse clinical syndromes including primary cutaneous cryptococcosis, often in non-immunocompromised hosts [8].

Molecular typing of clinical and environmental *C. neoformans* isolates from Europe using multilocus sequence typing (MLST) covering parts of six housekeeping genes and an intergenic spacer suggests different trajectories of clinical isolates from both varieties cultivated in Europe, i.e., mostly clonal expansion of *C. neoformans* var. *grubii* versus recombination of *C. neoformans* var. *neoformans* [6,9,10]. Previous studies did not find a correlation between genotypes, as assessed by MLST and clinical presentation [10]. Correlation between genotype and fungal phenotype is increasingly being recognized and may be responsible for clinical presentation and outcome of patients with cryptococcosis. However, isolates of the same genotype, as determined by MLST may dramatically differ in virulence traits, suggesting that typing methods with higher resolution might be needed to understand genetic determinants of virulence and clinical presentation [4,11]. In addition, isolates of different genotypes may show comparable virulence determinants as identified by infection models that are associated with the clinical presentation of cryptococcosis. 

Here, we studied *C. neoformans* var. *neoformans* isolates from patients with either disseminated or localized skin infections diagnosed in Germany and an environmental isolate from Germany to compare virulence factors and fungus–host interaction in two alternative host models to document phenotypic diversity of these fungi and screen for factors associated with different clinical presentations.

## 2. Materials and Methods

### 2.1. Cryptococcus Isolates and Reference Strains

Selected *C. neoformans* var. *neoformans* isolates from human cryptococcosis cases, one isolate per patient and an environmental isolate cultivated between 2013 and 2019 were included (Table 1). Isolates were provided by a network of laboratories throughout Germany. They were selected based on the availability of information needed to identify disease presentation, i.e., isolation from a skin infection with negative or minor serum antigen for localized infection or isolation from blood culture or cerebrospinal fluid (CSF) for disseminated infection. 

Fungi were obtained from frozen stocks (Microbank™, Pro-Lab Diagnostics Inc., Birkenhead, UK) and transferred to yeast peptone dextrose (YPD) agar using a sterile disposable inoculation loop. They were cultivated at 30 °C and stored at 4 °C until needed. For subsequent experiments, isolates and reference strains from overnight cultures (YPD at 25 °C with moderate shaking (130 rpm)) were centrifuged (1000× *g*, 10 min) and washed three times with phosphate-buffered saline (PBS; pH 7.2). The cell concentration was determined by using a hemocytometer and adjusted to the required cell concentration.

The reference strains *C. neoformans* var. *neoformans* B3501, a capsule deficient strain *C. neoformans* var. *neoformans* cap67, and *C. neoformans* var. *grubii* H99 were used in the *Galleria mellonella* infection model to control for the model’s ability to detect differences in virulence. Both *C. neoformans* var. *neoformans* reference strains were used in coincubation with *Acanthamoeba castellanii* to identify the ability to detect differences in uptake and cytotoxicity in the amoeba coincubation model. The *C. gattii* VGII strains R265 and CBS7750 were used as controls for determination of a hypermutator phenotype [12]. 

Fungal DNA for genotyping was extracted from overnight cultures in YPD cultivated at 25 °C with moderate shaking (130 rpm) using the Master Pure yeast extraction kit (LGC Lucigen, Middleton, WI, USA) with additional bead beating as described previously [5]. The genotype VNIV of the isolates was confirmed by a PCR assay targeting the *STR1* gene as previously described [13]. The mating-type was determined by specific PCR assays targeting the *STE12a* gene. MLST was performed according to the ISHAM standard protocol [14]. Allele and sequence types were assigned using the ISHAM MLST database. Strain and sequence information were submitted to this database. 

All media were prepared at the Robert Koch Institute in Berlin, Germany. Appropriate performance was checked with reference organisms. 

All experiments were performed and analyzed by an investigator (Z.M.B.) blinded to clinical information of the isolates.

### 2.2. Growth of C. neoformans var. neoformans at Different Conditions

The *C. neoformans* var. *neoformans* cells were harvested from overnight cultures in the dark and adjusted to a concentration of 1 × 10^6^ cells/mL in 0.85% NaCl solution. A serial dilution in 0.85% NaCl was performed and 5 µL plated on yeast nitrogen base (YNB) agar. 

Cells were incubated at 30 °C for 48 h in the dark at different concentrations of H_2_O_2_ (0, 5, 10 µM, 1 mM), different carbon dioxide concentrations (0 vs. 5.0% CO_2_) and different pH (5 vs. 7). The effect of temperature was determined by incubation at 25 °C, 30 °C, or 37 °C. The effect of each condition on the cell growth was determined by counting the number of colony-forming units (CFU) after the incubation. Tests were performed in three independent replicates with five technical replicates each. 

### 2.3. Expression of Virulence Factors

For quantification of specific virulence factors, cells from overnight cultures were washed and adjusted to a concentration of 1 × 10^6^ cells/mL in 0.85% NaCl solution. 

Melanization was determined by spotting 5 µL of suspension on Niger Seed agar and incubating at 30 °C for 48 h in the dark. Plates were photographed and converted to black and white pictures. Pigmentation was quantified digitally (ZEN2012, Carl Zeiss Microscopy GmbH, Jena, Germany) by measuring the pixel intensity. The pixel intensity was normalized by dividing the intensity by the sum of all measured pixels. Therefore, lower values of normalized pixel intensity indicate stronger melanization. Three individual experiments with four technical replicates were performed.

$$pixel\;itensity\;\left(norm.\right)=\frac{pixel\;itensity\;\left(single\;colony\right)}{\Sigma \left(all\;measured\;pixels\right)}$$

Urease activity was assessed using Christensen’s urea agar. Fungal suspensions (5 µL of 1 × 10^6^ cells/mL) were spotted in the middle of an agar plate and incubated at 30 °C for 48 h. The hydrolysis of urea catalyzed by urease leads to a pH shift which can be detected by the pH indicator in the agar. The diameter of the resulting halo was measured digitally (ImageJ version 1.52a, [15]) in horizontal and vertical directions. Three individual experiments were performed.

Phospholipase activity was determined by spotting 5 µL of suspension in the middle of egg yolk agar and incubating at 30 °C for 120 h. The resulting precipitation halo was measured and the phospholipase activity (*Pz* value) was calculated following the principle described by Price et al. by measuring the ratio of the colony’s diameter to its surrounding precipitation halo [16]. Therefore, a *Pz* value of 1.0 indicates that the tested isolate was phospholipase negative. Three individual experiments were performed.

$$Pz=\frac{colony^{\prime }s\;diameter}{halo^{\prime }s\;diamter}$$

Capsule and cell sizes were measured after cultivation in minimal medium (15 mM dextrose, 10 m MMgSO_4_, 29.4 mM KHPO_4_, 13 mM glycine, and 3 μM thiamine, pH 5.5 [17]) at 30 °C for 24 h at moderate shaking (130 rpm) to induce capsule formation. Twenty µL of suspension was stained with 80 µL India ink., viewed under a microscope (40× magnification) and imaged via moticam (Motic, Xiamen, China). Capsule, cell body and total cell sizes were measured digitally (ImageJ version 1.52a, [15]) in horizontal and vertical directions for 20 randomly selected cells. 

The ability to spontaneously develop resistance to 5-fluorocytosine (5-FC) was used as an indicator of mutation frequency as a nonsense mutation in the DNA repair gene *MSH2* favors the formation of spontaneous mutations [12]. The spontaneous 5-FC resistance was measured following the principle described before [12]. In short, fungal suspensions (100 µL of 1 × 10^6^ cells/mL) were incubated on YNB agar with and without 100 µg/mL 5-FC (Sigma-Aldrich, Burlington, MA, USA) at 30 °C for 48 h. Resistance to 5-FC was measured by counting the number of CFU on the plates. The hypermutator strain CBS7750 and the *C. gattii* R265 without this phenotype were used as controls. Three individual experiments were performed [12].

### 2.4. Interaction of Cryptococcus with Acanthamoeba

*A. castellanii* 1BU (ATCC PRA-105, Manassas, VA, USA) was obtained from a stock culture maintained at the Robert Koch Institute. Trophozoites were incubated at 29 °C with 4.5% CO_2_ in 20 mL peptone yeast glucose medium (PYG; ATCC 712, Manassas, VA, USA) containing 1% (*v*/*v*) of Penicillin/Streptomycin. Amoebas were passaged weekly and one day before each experimental unit by transferring the culture into a new cell culture flask with filter cap and diluting the culture 1:10 with fresh medium. 

Phagocytosis by and cytotoxicity for *A. castellanii* was determined following the procedure described before by Steenbergen et al. [18]. In brief, phagocytosis indices were determined by coincubation of fungi (2 × 10^5^) and amoeba (1 × 10^5^) in 100 µL PYG medium at 29 °C and 4.5% CO_2_ for two hours in the dark. Cells were stained, viewed by microscope and the ratio of amoebas with engulfed yeast cells was calculated. For determination of cytotoxicity, fungi (1 × 10^4^) and amoeba (1 × 10^4^) were coincubated in 100 µL PBS at 29 °C and 4.5% CO_2_ for 48 h in the dark. The trypan blue exclusion assay (Sigma-Aldrich, Burlington, MA, USA) was performed to determine the percentage of dead amoebas [19]. As negative control, amoebas were incubated alone in PBS. The reference strain B3501A and the acapsular strain cap67 were used to determine the detectable range of uptake and cytotoxicity of the coincubation experiments. For each experimental setup, three individual experiments were performed with eight technical replicates each.

### 2.5. Infection of Galleria mellonella Larvae

Larvae of *G. mellonella* were obtained in a shop for fishing supplies (Angelbedarfsladen Malchow, Berlin, Germany) and experiments were started immediately after obtaining the larvae. One day before inoculation, animals were weighed and collected in experimental groups of 20. The larvae were divided into equal weight groups (250–600 mg) and weights were visualized via boxplots to obtain a cross-section of the total population but equally distributed between the groups to ensure comparability and thereby exclude body volume as a determining factor. Larvae with dark spots were excluded because melanization might be evidence for infection [20]. The animals were incubated overnight at 37 °C in Petri dishes (Ø = 10 cm) containing wood chips before inoculation. 

*Cryptococcus* cells were harvested from overnight cultures, washed and adjusted to a concentration of 0.5 × 10^8^ cells/mL in 0.85% NaCl. The cell suspensions were carefully vortexed and taken up by an insulin syringe. Larvae were disinfected using a cotton swab containing 70% ethanol and inoculated through injection of 20 µL into the last left proleg. Each animal was infected with 1 × 10^6^ cells. The inoculum was controlled by plating (5 µL) as a serial dilution on YPD and brain heart infusion (BHI) agar. In each experiment, control groups were used to estimate a traumatic effect of inoculation (injection of 20 µL sterile 0.85% NaCl), to estimate the virulence potential of unviable *C. neoformans* var. *neoformans* cells (H99 inactivated at 60 °C for one hour), and to monitor for larval viability (no intervention). Larvae were incubated at 37 °C in Petri dishes containing wood chips and controlled for survival (movement upon stimulus) every day. Petri dishes with fresh wood chips were changed daily. 

### 2.6. Statistical Analysis

Experiments studying the effect of incubation conditions on the fungal growth were analyzed by performing a two-way ANOVA and additional Tukey’s multiple comparison test.

For differences in virulence factor phenotype, datasets were first checked for a normal distribution. For this purpose, the Kolmogorov–Smirnov test (if *n* < 50) or Shapiro–Wilk test (if *n* > 50) was performed, and the test results were visually confirmed using QQ-plots. Next, the residuals and variances were checked for equal distribution. Therefore, a visual inspection of plots was made, and an F-test or Bartlett’s test was performed, depending on whether two or more groups were compared. If the data were normally distributed and the residuals and variances were equal, an unpaired t-test was performed when comparing two groups, or an ordinary one-way ANOVA when comparing several groups. If the variances were not equal, the respective tests were adjusted by a Welch correction. If the datasets did not have a normal distribution of the data, a Wilcoxon–Mann–Whitney test was performed to compare two groups and a Kruskal–Wallis test for the comparison of multiple groups. 

For *G. mellonella* survival analysis, larval survival was documented and pupated animals were censored on the day of pupation. Survival was displayed via Kaplan–Meier curves. For statistical analysis, differences of survival curves were tested for significance pairwise using a log-rank test. The median survival of larvae was calculated and analyzed similar to the virulence factors analysis. Statistical analysis was performed using Graphpad Prism 8-software (GraphPad Software, San Diego, CA, USA) and for all tests performed, the significance level was set at *p* < 0.05 in two-sided tests.

## 3. Results

### 3.1. Growth of Isolates Might Be Affected by Temperature

No differences in growth could be observed between the isolates at 30 °C and 37 °C. Incubation of some *C. neoformans* var. *neoformans* isolates (13-0492: environmental; 18-0352: disseminated infection, 19-1016: localized infection) displayed reduced growth at 25 °C compared to incubation at 30 °C (Supplemental Figure S1). Two strains from disseminated infections (19-0346 and 18-0352) showed less growth at 25 °C compared to 37 °C. Incubation at different H_2_O_2_ concentrations only affected one strain (19-0346) isolated from blood. The presence of 5% CO_2_ resulted in a smaller colony size for two isolates from disseminated infections (18-0352, 19-0346) and the environmental isolate.

### 3.2. Isolates Demonstrate Differences in Melanization, Capsule and Cell Size

Individual *Cryptococcus* isolates display significant differences (*p* ≤ 0.0001) for all virulence factors analyzed, including melanization (0.047–0.077 norm. pixel intensity), urease activity (7.9–33.2 mm), phospholipase activity (*Pz* values between 0.31 and 0.64), capsule (2.4–3.8 μm), cell body (3.8–6.6 μm) and total cell size (6.2–10.4 μm). The environmental isolate is comparable to the clinical isolates for all virulence factors with intermediate values between those of the clinical isolates. 

**Figure 1:**
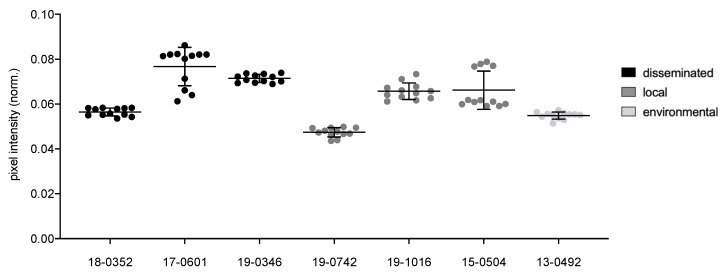
Melanization of *Cryptococcus neoformans* var. *neoformans* isolates. 5 µL containing 10^6^ cells were spotted on Niger seed agar and incubated at 30 °C for 48 h. Plates were photographed and converted to black/white pictures. The pixel intensity of the colonies was measured digitally. Measurements were normalized by division through the sum of all measured pixels. Therefore, lower values of normalized pixel intensity indicate stronger melanization. Black lines show means with standard deviation of three biological replicates with five measurements each. Dots show individual data points.

**Figure 2:**
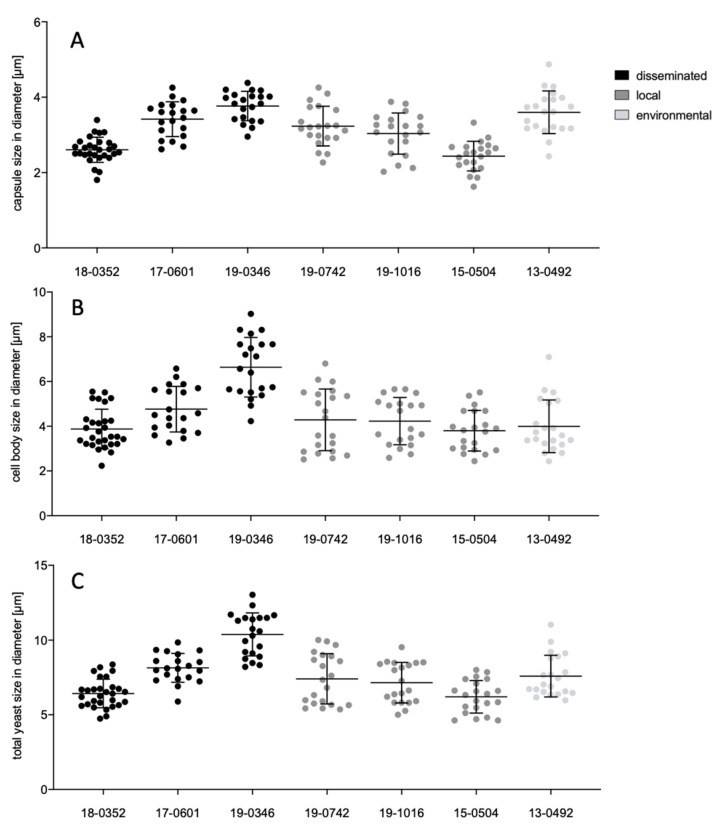
Capsule (**A**), cell body (**B**) and total cell size (**C**) of *Cryptococcus neoformans* var. *neoformans* isolates from disseminated infections (black), localized soft tissue infections (grey), and the environment (light grey). Cells were incubated in minimal medium at 30 °C for 24 h. Cells were stained with India ink and measured digitally. Black lines show means with standard deviation of 20 measured cells per isolate. Dots show individual data points.

When isolates from different clinical presentations were pooled, those from disseminated infections presented a stronger melanization (*Pz* values of 0.07 vs. 0.06; *p* = 0.0037; Figure 1), larger capsules (3.15 µm vs. 2.92 µm; *p* = 0.0164), larger cell bodies (4.33 µm vs. 3.79 µm; *p* = 0.0022) and total cell sizes (7.98 µm vs. 6.59 µm; *p* = 0.0164; Figure 2) as compared to isolates from localized infections. Pooled isolates from disseminated infections presented a higher diversity in cell body size (std. deviation 1.6 µm) and total cell size (std. deviation 2 µm). In contrast, isolates from localized infections presented comparable cell bodies and total cell sizes (std. deviation of 1.1 µm and 1.5 µm, respectively). 

**Figure 3:**
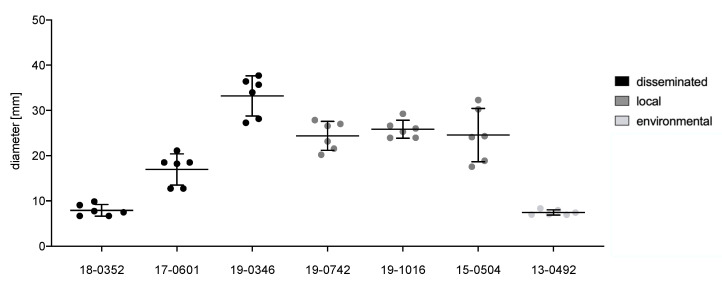
Urease activity of *Cryptococcus neoformans* var. *neoformans* isolates from disseminated infections (black), localized soft tissue infections (grey), and the environment (light grey). A total of 5 µL containing 10^6^ cells were spotted on Christensen’s urea agar and incubated at 30 °C for 48 h. Halos surrounding colonies were measured digitally. Black lines show means with standard deviation of three biological replicates with two measurements each.

**Figure 4:**
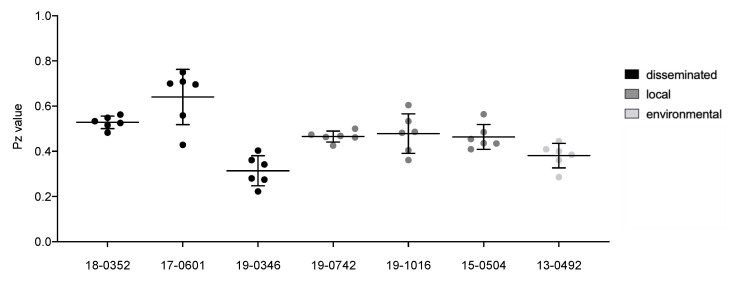
Phospholipase activity of *Cryptococcus neoformans* var. *neoformans* isolates from disseminated infections (black), localized soft tissue infections (grey), and the environment (light grey). A total of 5 µL containing 10^6^ cells were spotted on egg yolk agar and incubated at 30 °C for five days. Halos surrounding colonies were measured digitally. Black lines show means with standard deviation of three biological replicates with two measurements each. Dots show individual data points.

While individual isolates differed in urease and phospholipase activity, no significant differences between cells from disseminated and localized infections could be observed after the pooling of isolates from different clinical presentations (Figure 3 and Figure 4). However, isolates from disseminated infections showed greater variability in urease activity compared to isolates from local infections (std. deviation of 11.2 mm and 3.8 mm, respectively). Measurements of phospholipase activity displayed similar results with a standard deviation of *Pz* = 0.16 for disseminated isolates and *Pz* = 0.06 for localized isolates. 

Cultivation of CBS7750 with a hypermutator phenotype resulted in 49 CFU on 5-FC containing plates, while R265 did not show growth as expected. Interestingly, only the *C. neoformans* var. *neoformans* environmental isolate produced 33 CFU on 5-FC containing plates suggesting a hypermutator phenotype. In contrast, all clinical isolates formed no or few CFU and thus did not differ significantly from the reference isolate R265. The isolate 19-1016 could not be examined with this method as it is resistant to 5-FC.

### 3.3. Isolates from Disseminated Infections Demonstrate Higher Uptake and Cytotoxicity for Acanthamoeba 

**Figure 5:**
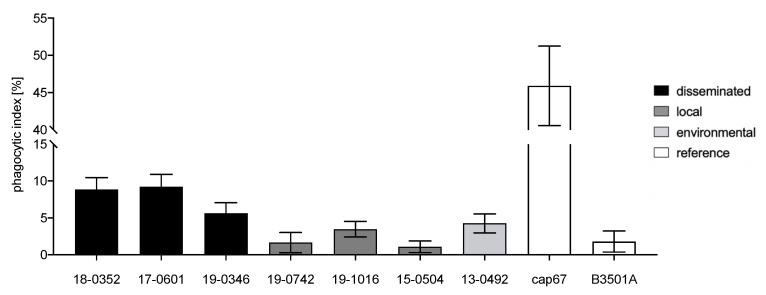
Phagocytic index representing the percentage of *Acanthamoeba castellanii* with intracellular *Cryptococus neoformans* var. *neoformans* cells after incubation in PYG medium at 29 °C for two hours. Clinical isolates from disseminated infections (black) are better phagocytosed than isolates from localized infections (grey) and the environment (light grey). To define the range of this assay, a *C. neoformans* wild-type reference strain (B3501A) and an acapsular strain (cap67) were included (white). The standard deviations of three independent experiments with eight technical replicates each are shown.

Coincubation of *C. neoformans* var. *neoformans* with *A. castellanii* resulted in significantly different phagocytosis indices with *C. neoformans* var. *neoformans* reference strain B3501A and acapsular mutant strain cap67 defining the detectable range of the method performed (*p* < 0.0001; Figure 5). While almost all *C. neoformans* var. *neoformans* isolates resulted in a higher phagocytic index than B3501A (1.8%), they all demonstrate significantly lower phagocytic indices than the strain cap67 (45.9%) lacking the antiphagocytic capsule.

Cryptococci isolated from disseminated infections showed a significantly higher mean phagocytic index as compared to isolates from localized soft tissue infections (7.9 vs. 2.1%; *p* < 0.0001). The environmental isolate resulted in a phagocytic index between cells from disseminated and localized cells. 

**Figure 6:**
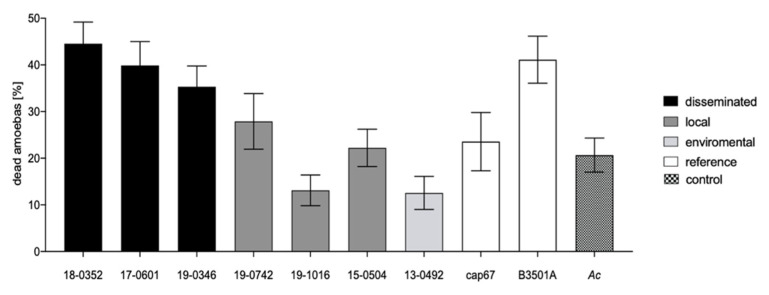
Cytotoxicity of *Cryptococcus neoformans* var. *neoformans* isolates for *Acanthamoeba castellanii* after incubation in PBS medium at 29 °C for 48 h. Clinical isolates from disseminated infections (black) are more cytotoxic for amoebas than isolates from localized soft tissue infections (grey) and the environment (light grey). To define the range of this assay, a wild-type reference strain and an acapsular strain were included (white). *A. castellanii* was incubated without fungi as control (checkered). Standard deviations of three independent experiments with eight technical replicates each are shown.

The coincubation of *C. neoformans* var. *neoformans* with *A. castellanii* for 48 h resulted in significantly different percentages of dead amoebas (*p* < 0.0001; Figure 6). The reference strain B3501A had the second-highest cytotoxicity for amoeba, resulting in a mean percentage of dead amoebae of 41%. The capsule mutant strain, on the other hand, did not affect amoeba viability and is comparable to incubation of amoeba alone (24% and 21%, respectively). The environmental strain and one strain from a localized infection (19-1016) even had a positive effect on amoeba viability, as coincubation resulted in a lower percentage of dead amoebae than incubation of *A. castellanii* alone (13% vs. 21%). 

Taken together, cells from disseminated infections showed higher cytotoxicity for amoeba than isolates from localized infections (40% vs. 21%; *p* < 0.0001). 

### 3.4. Median Survival of G. mellonella Larvae Varies Significantly between Isolates 

**Figure 7:**
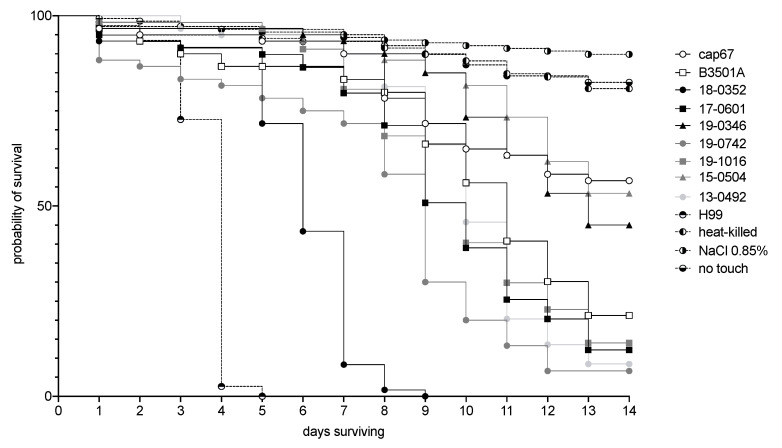
Virulence of *Cryptococcus neoformans* var. *neoformans* isolates in the *Galleria mellonella* injection model. Kaplan–Meier survival curves demonstrate that isolates recovered from human infections, either disseminated infections (black), localized soft tissue infections (grey), or environmental isolates (light grey), show substantial differences in virulence. While all *C. neoformans* var. *neoformans* isolates show longer median survival compared to *C. neoformans* var. *grubii* H99, they show shorter median survival than cap67, a strain lacking a capsule that was demonstrated to be avirulent in mice (40, 41). Each symbol represents a tested isolate; dashed lines represent control groups. Each curve represents cumulative data of three biological replicates with 20 larvae per group and experimental unit.

*C. neoformans* var. *grubii* H99 showed the highest virulence in *G. mellonella* and *C. neoformans* var. *neoformans* cap67 the lowest (median 4 vs. 17 days; Figure 7). Infection with clinical and the environmental *C. neoformans* var. *neoformans* strains showed median survival between 6 and 13.5 days (*p* = 0.0047) with all isolates being less virulent than H99. The environmental isolate resulted in a median survival of 10 days.

Curve comparison after pooling of isolates revealed significantly faster larval killing after infection with cells from disseminated infections compared to isolates from localized infection (*p* = 0.0276). However, the median survival of the two groups of isolation was not statistically different (9 days vs. 10 days). 

The most virulent strain in *G. mellonella* (18-0352), isolated from a disseminated infection, also demonstrated a high phagocytic index in coincubation with *Acanthamoeba* and the highest cytotoxicity for the amoeba. In contrast, isolate 19-0346, a mating-type isolate cultivated from a disseminated infection, demonstrated a high phagocytic index and high cytotoxicity for amoeba and was among the least virulent in the *G. mellonella* infection model. 

## 4. Discussion

*C. neoformans* var. *neoformans* isolates cultivated in Germany show a remarkable phenotypic diversity including capsule size, production of melanin, urease and phospholipase activity. This may impact the differences in uptake into phagocytic amoebae, cytotoxicity for amoebae and virulence in the *G. mellonella* infection model. Isolates from disseminated infections showed larger capsules, cell bodies and higher melanin production as compared to isolates from localized skin infections. Although isolates from disseminated infections showed variable degrees of urease and phospholipase production, they demonstrated higher uptake into amoeba and cytotoxicity and shorter survival of *G. mellonella*. 

Epidemiologic studies performed in the context of disseminated infections by *C. neoformans* var. *grubii* in HIV-infected patients demonstrate that fungal genotype may affect clinical outcomes [4]. It was suggested that cell surface changes, including capsule size, melanization and changes in cell size, i.e., large titan cells and small micro cells are phenotypic changes that correlate with virulence potential when environmental fungi enter hosts [4]. It was demonstrated that isolates from human cryptococcosis patients, mainly *C. neoformans* var. *grubii* and *C. gattii*, show substantial variation in virulence in infection models including the *G. mellonella* infection model [21,22]. 

So far, few studies have been performed to correlate the virulence potential of *C. neoformans* var. *neoformans* and the clinical presentation of these infections. *C. neoformans* var. *neoformans* are the second most prevalent agents of cryptococcosis in Europe [5,6]. In addition, they can be cultivated from environmental sources including trees, dust, pigeon droppings and animals including cats, cows and pigeons [9]. Based on environmental sampling from non-random sites, it was suggested that this fungus may be adapted to colder, wetter climates currently found in central Europe [10]. However, a recent report on the cultivation from Saudi Arabian desert soil suggests that this fungus is more widespread and potentially more adaptable to various environmental conditions than previously thought [23]. Interestingly, the population structure of *C. neoformans* var. *neoformans* isolates from Europe shows high genotypic diversity potentially facilitated by sexual reproduction [9]. As this process may generate phenotypic diverse offspring, laboratory tests might be needed address to virulence potential and therefore the public health potential of *C. neoformans* var. *neoformans* isolates.

Different models were used to study fungal virulence. Amoebas are a relatively easy and cheap model system. They have gained interest as some aspects of the interaction between amoeba and fungi resemble the interaction between fungi and phagocytic cells. The capsule, melanin production and phospholipase activity were shown to be important for the survival of *Cryptococcus* in coincubation with amoeba, while urease activity and mating-type were not. This model does not reflect other disease determinants such as immune-mediated host damage, an important part of animal cryptococcosis [24]. However, the significant differences in uptake by amoeba and cytotoxicity suggest that this model may be used to screen for the potential of strains to cause disseminated infections.

*G. mellonella* has emerged as a model system to explore fungal virulence and pathogenesis. *G. mellonella* and the mammalian immune system share some similarities including pathogen recognition, inflammatory responses, phagocytosis of pathogens and phagocyte responses to fungal uptake [25].

Primary cutaneous cryptococcosis typically presents as a solitary skin infection site after transcutaneous injury with a contaminated source. These infections are thought to be mostly caused by *C. neoformans* var. *neoformans* (genotype VNIV) isolates and generally have a favorable outcome without systemic signs of infection and mostly negative serum antigen. They are often diagnosed in patients without underlying immunosuppression [8]. The absence of dissemination in these cases might be a consequence of the host’s immune system and fungal attributes. Genotype VNIV strains were shown to be less virulent in animal models than VNI strains. In addition, cryptococci with the mating-type are less virulent in infection models and this was confirmed for *C. neoformans* var. *neoformans* [7,26]. 

Nevertheless, even Da strains may cause disseminated infections despite low virulence potential in infection models as demonstrated here by the isolate 19-0346 from a disseminated infection and low virulence in *G. mellonella*. This has previously been documented for another Da isolate from a disseminated infection in an AIDS patient and low virulence in a mouse model [7].

Previous studies using molecular typing (MLST) did not show that specific genotypes account for primary or secondary skin or disseminated infections [10]. Our results suggest that phenotypic traits and non-vertebrate infection models may help to identify isolates with the potential to cause disseminated infections. This seems to be important, given the occurrence of a genetically distinct population of *C. neoformans* var. *neoformans* in the environment in Europe that recombines sexually and asexually and therefore may produce offspring with different virulence potential [10]. 

Interestingly, the only environmental isolate we included showed evidence for a hypermutator phenotype. This phenotype can be caused by mutations in mismatch repair proteins. It may provide a mechanism for the rapid creation of phenotypic diversity and could also be demonstrated to be of clinical relevance, i.e., be associated with relapsed disease and antifungal drug resistance [27]. 

## 5. Conclusions

This report documents substantial phenotypic variability of *C. neoformans* var. *neoformans* isolated in Germany. The differences of strains from localized and disseminated infections in interaction with amoeba are in line with the importance of host cells in the pathogenesis of disseminated infections. Phenotypic traits and non-vertebrate infection models may help to understand the virulence potential of *Cryptococcus* isolates. The use of *C. neoformans* var. *neoformans* strains from patients with localized and disseminated infections may offer an alternative approach to study mechanisms of fungal dissemination. Both infection models may help to screen *C. neoformans* potential to cause disseminated infections.

## Figures and Tables

**Table 1:** *C. neoformans* var. *neoformans* strains isolated in Germany used in this study.

Strain Identifier	Isolation Site	Serum Antigen	Mating-Type	Sequence Type
13-0492	pigeon guano	not applicable	a	524
15-0504	skin	negative	α	597
17-0601	CSF	unknown	α	270
18-0352	CSF	unknown	α	116
19-0346	blood	unknown	a	661
19-0742	skin	negative	α	567
19-1016	skin	1:32	α	514

## Data Availability

All data are available in graphs and tables in the manuscript and supplement data.

## Supplementary Materials

The following supporting information can be downloaded at: https://www.mdpi.com/article/10.3390/microorganisms10020321/s1, Figure S1: Effect of temperature on growth of *Cryptococcus neoformans* var. *neoformans*.
