# Supplementary material for: Phenotypic Diversity of Cryptococcus neoformans var. neoformans Clinical Isolates from Localized and Disseminated Infections
Source: Microorganisms. 2022 Jan 29;10(2):321. doi: 10.3390/microorganisms10020321 (PMC8878387; doi:10.3390/microorganisms10020321)
Supplement: Supplementary file 1 [file microorganisms-10-00321-s001.zip › microorganisms-1564988-supplementary.pdf]

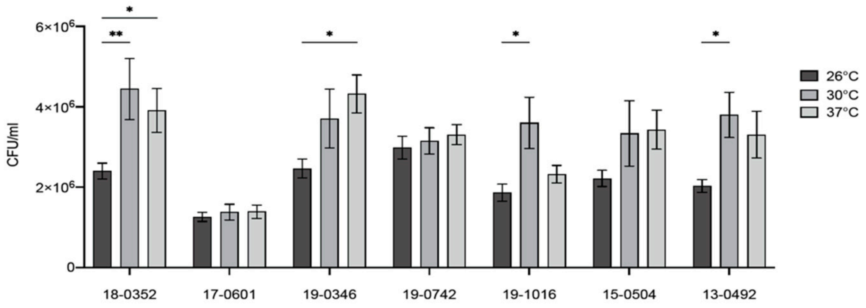

Supplemental Figure S1. Effect of temperature on growth of *C. neoformans var. neoformans*. Isolates from disseminated infections (n=3), localized soft tissue infections (n=3), or the environment (n=1). Cells were spotted in 10-fold serial dilutions (starting concentration of 1×10^6 cells/ml) on YNB agar and incubated at 26°C, 30°C or 37°C in the dark. Cell growth was determined by counting the number of colony-forming units after incubation for 48 hours. Black lines show the standard deviations of three independent replicates with five technical replicates each. Asterisks indicate statistical significance with *p<.05, **p <.01.
